# Combined Genomic, Transcriptomic, Proteomic, and Physiological Characterization of the Growth of *Pecoramyces* sp. F1 in Monoculture and Co-culture With a Syntrophic Methanogen

**DOI:** 10.3389/fmicb.2019.00435

**Published:** 2019-03-06

**Authors:** Yuanfei Li, Yuqi Li, Wei Jin, Thomas J. Sharpton, Roderick I. Mackie, Isaac Cann, Yanfen Cheng, Weiyun Zhu

**Affiliations:** ^1^Laboratory of Gastrointestinal Microbiology, National Center for International Research on Animal Gut Nutrition, Nanjing Agricultural University, Nanjing, China; ^2^Joint International Research Laboratory of Animal Health and Food Safety, Nanjing Agricultural University, Nanjing, China; ^3^Department of Microbiology – Department of Statistics, Oregon State University, Corvallis, OR, United States; ^4^Department of Animal Sciences, University of Illinois at Urbana-Champaign, Champaign, IL, United States; ^5^Department of Microbiology, University of Illinois at Urbana-Champaign, Champaign, IL, United States; ^6^Division of Nutritional Sciences, University of Illinois at Urbana-Champaign, Champaign, IL, United States; ^7^Carl R. Woese Institute for Genomic Biology, University of Illinois at Urbana-Champaign, Champaign, IL, United States

**Keywords:** anaerobic fungus, methanogen, metabolism, genome, RNAseq, iTRAQ

## Abstract

In this study, the effects of a syntrophic methanogen on the growth of *Pecoramyces* sp. F1 was investigated by characterizing fermentation profiles, as well as functional genomic, transcriptomic, and proteomic analysis. The estimated genome size, GC content, and protein coding regions of strain F1 are 106.83 Mb, 16.07%, and 23.54%, respectively. Comparison of the fungal monoculture with the methanogen co-culture demonstrated that during the fermentation of glucose, the co-culture initially expressed and then down-regulated a large number of genes encoding both enzymes involved in intermediate metabolism and plant cell wall degradation. However, the number of up-regulated proteins doubled at the late-growth stage in the co-culture. In addition, we provide a mechanistic understanding of the metabolism of this fungus in co-culture with a syntrophic methanogen. Further experiments are needed to explore this interaction during degradation of more complex plant cell wall substrates.

## Introduction

In the rumen, microorganisms, which are mainly composed of anaerobic fungi, bacteria, archaea, and protozoa, have coevolved for millions of years, making the rumen one of the most effective and highly evolved systems regarding degradation of recalcitrant lignocellulosic plant material in nature (Russell and Rychlik, 2001; Weimer et al., 2009). Within this system, the diverse microbial communities cooperate efficiently in the digestion and conversion of plant biomass in feeds to various compounds crucial for body maintenance and performance (Kim et al., 2011; Mao et al., 2016). Anaerobic fungi, bacteria, and protozoa degrade and ferment ingested plant biomass and release hydrogen during this process (Akin et al., 1988). However, the accumulation of hydrogen is energetically unfavorable and can inhibit the fermentation of ingested feed (Ungerfeld, 2015). Ruminal methanogens are effective hydrogen utilizers and can use the hydrogen generated to reduce carbon dioxide (which is also a product of primary fermentation) to methane, thereby keeping the steady-state hydrogen concentration low and the rumen operating more efficiently (Janssen and Kirs, 2008). Thus, trophic interactions exist between the methanogenic archaea and the hydrogen-producing microorganisms that includes both anaerobic fungi and bacteria.

Anaerobic fungi assigned to the phylum Neocallimastigomycota play key roles in the decomposition of recalcitrant plant lignocellulosic materials in the rumen. Since the identification of anaerobic fungi by Orpin (1975), 11 genera assigned to the phylum Neocallimastigomycota have been described: *Neocallimastix* (Heath et al., 1983), *Caecomyces* (Gold et al., 1988), *Piromyces* (Gold et al., 1988), *Orpinomyces* (Barr et al., 1989), *Anaeromyces* (Breton et al., 1990), *Cyllamyces* (Ozkose et al., 2001), *Buwchfawromyces* (Callaghan et al., 2015), *Oontomyces* (Dagar et al., 2015), *Pecoramyces* (Hanafy et al., 2017), *Feramyces* (Hanafy et al., 2018), and *Liebetanzomyces* (Joshi et al., 2018). Despite their potent capacities for lignocellulose degradation, anaerobic fungi and their enzymes are yet to be exploited in biotechnological processes. This is largely due to their obligately anaerobic lifestyle and a poor understanding of their growth requirements and metabolic characteristics. Anaerobic fungi can ferment a wide range of fermentable sugars, such as glucose, fructose, xylose, and cellobiose as energy sources. These are utilized to produce H_2_, CO_2_, formate, acetate, lactate, and ethanol as the major fermentation end products (Lowe et al., 1987; Teunissen et al., 1993). In their natural habitat in the rumen and hind-gut of large mammalian herbivores, anaerobic fungi grow together in communities with other microbes. Anaerobic fungi and closely associated methanogens can be isolated from mixed microbial communities and can be cultured in stable co-culture in media that do not contain appreciable amounts of compounds that methanogens need to grow (Cheng et al., 2009). Anaerobic fungal-methanogen co-cultures have been shown to be stable with robust growth evident over long periods of time (Bauchop and Mountfort, 1981; Cheng et al., 2009). Additionally, in co-cultures, as a consequence of inter-species hydrogen transfer, the metabolite profile of the anaerobic fungus alters, shifting away from more reduced products, such as lactate and ethanol, toward acetate and formate. The formate and hydrogen, end products of fungal fermentation, are used by the methanogens to produce methane (Cheng et al., 2009; Jin et al., 2011; Li et al., 2016). Meanwhile, the fiber-degrading ability of the anaerobic fungus in co-cultures was improved (Jin et al., 2011). Thus, the metabolic profile of anaerobic fungi in the co-culture is comparable to that of their counterparts in the rumen, where hydrogen and formate are known to be transient and low (Hungate, 1967; Hungate et al., 1970), and the fiber-degrading ability is known to be high (Krause et al., 2003). Thus, investigating the interaction between anaerobic fungi and co-cultured methanogen might provide insights into the complex microbial interactions in the rumen.

In recent years, omics-based techniques have been used to study the diversity, ecology, and biology of anaerobic fungi. Five genomes of anaerobic fungal strains have been reported, including *Piromyces* sp. E2, *Pecoramyces ruminantium* C1A, *Anaeromyces robustus, Neocallimastix californiae*, and *Piromyces finnis* (Youssef et al., 2013; Haitjema et al., 2017). The transcriptomes of *Pecoramyces ruminantium* C1A, *Piromyces finnis, Neocallimastix californiae, Caecomyces churrovis, Anaeromyces mucronatus, Neocallimastix frontalis, Orpinomyces joyonii, Piromyces rhizinflata*, and *Anaeromyces robustus* have been described (Couger et al., 2015; Solomon et al., 2016; Henske et al., 2017; Gruninger et al., 2018). To our knowledge, there are no studies that apply functional genomic, transcriptomic, and proteomic approaches to interrogate the effect of co-culturing a methanogen on the metabolism, including expression of fiber-degrading enzymes, of an anaerobic fungus.

In the present study, we used genomic, transcriptomic, and metabolomic data of the anaerobic fungal monoculture to draw a metabolic pathway of the fungus. The mRNA expression profile of the anaerobic fungus *Pecoramyces* sp. F1 in the presence and absence of its syntrophic methanogen, *Methanobrevibacter thaueri*, was also investigated. By combining the foregoing analysis with the anaerobic fungal proteome dynamics and analysis of the metabolites induced by growth with the methanogen, we reveal the effects of the archaeon on the metabolism of the anaerobic fungus.

## Materials and Methods

### Maintenance of Anaerobic Fungal Monoculture and Co-culture

The anaerobic fungus *Pecoramyces* sp. F1, formerly described as *Piromyces* sp. F1, and its symbiotic methanogen, *Methanobrevibacter thaueri*, were isolated and identified from goat rumen by Jin et al. (2011). The culture was maintained in rumen fluid media (Davies et al., 1993) with 1% (w/v) rice straw as substrate and transferred every 3 days. The media was prepared according to Cheng et al. (2009) and 90 ml media was dispensed into 160 ml serum bottle with 1 g rice straw as substrate. At each transfer, 10 ml of 3-day-old culture was inoculated into 90 ml of fresh media and incubated at 39°C for 3 days. The fungal monoculture was obtained by adding chloramphenicol (50 mg l^-1^ final concentration) to inhibit the growth of the associated methanogen (Cheng et al., 2009). The relative abundance of methane in the head-space gas of the monoculture was analyzed by GC-TCD (Agilent 7890B, Agilent, Santa Clara, CA, United States) to ensure that no methane was being produced by the culture to confirm that the methanogen was no longer present in fungal pure culture studies.

### Experimental Design and Sample Collection

In the current study, the medium used for experiments was a modified medium M2 (Barichevich and Calza, 1990) with 2.16 g l^-1^ (12 mM) glucose as substrate. The medium was prepared and dispensed under anaerobic conditions into serum bottles (90 ml/bottle), with pH adjusted to 6.8 (Li et al., 2016). For anaerobic fungal genome sequencing, 40 bottles of *Pecoramyces* sp. F1 monoculture were incubated at 39°C for 72 h without shaking. The fungal cells were then harvested by centrifugation at 10,000 × *g* for 15 min.

To investigate the effects of co-culturing with *M. thaueri* on the metabolism of *Pecoramyces* sp. F1, the anaerobic fungus was grown alone (monoculture) and also in co-culture with the methanogen at 39°C without shaking. A total of 72 bottles were used for the experiment; details of the protocol information are shown in Supplementary Figure 1. Samples were collected from each replicate for transcriptomic, proteomic, and metabolite analysis. The total volume of gas accumulated in each culture over the incubation period was also measured using the pressure transducer technique (Theodorou et al., 1994). After each reading, the head-space was vented to return the pressure to ambient conditions. Furthermore, the gas drawn was analyzed for CH_4_ and H_2_ content. Samples from the cultures were collected at approximately 50% and 100% of maximum gas production (i.e., mid- and late-growth stages) as determined from previously generated gas accumulation curves. The pH was measured at each time point immediately upon removing crimp-seals and stoppers from the serum vials. Aliquots of 5 ml supernatant were then collected and stored at -20°C for subsequent analysis of water-soluble metabolites. The rest of the culture was then centrifuged at 8,000 × *g* for 15 min, and 1 ml of supernatant was used for the analysis of residual glucose with a commercial glucose kit (Nanjing Jiancheng Biotechnology Institute, Nanjing, China). The cells from the remaining six bottles, representing each replicate, were then mixed and split into two parts for RNAseq and iTRAQ analysis. Two bottles of each replicate were used for the analysis of gas, glucose, pH, and water-soluble metabolites.

### DNA Extraction, Sequencing, Genome Assembly, and Gene Calling and Annotation

Genomic DNA was extracted from a 3-day-old anaerobic fungal monoculture with the CTAB method (Cheng et al., 2017). Briefly, the culture was centrifuged and ground in liquid nitrogen. CTAB buffer was added to dissolve the powder and phenol/chloroform/isoamyl alcohol (25:24:1) was then used to purify the DNA. Three libraries with insert sizes of 170 bp, 350 bp, and 6,000 bp were prepared at BGI (Beijing Genomics Institute, Shenzhen, China) according to the manufacturer’s instructions (Illumina). Paired-end sequencing was conducted on an Illumina Hiseq 2000 platform (BGI, Shenzhen, China). A total of 28.67 Gb in 159,302,966 quality-filtered paired-end reads were used for assembly (Supplementary Table 1). The quality-filtered reads were assembled with SOAPdenovo V1.05 (Li et al., 2008, 2010) using a kmer value of 43. The assembly was then optimized by the paired-end and overlap relationship of reads through mapping reads to assembled contigs. Gene calling was then conducted using a combination of Augustus V2.6.1 and Genemarkes V2.3e (Ter-Hovhannisyan et al., 2008; Keller et al., 2011). Transposable elements (TEs) were identified by RepeatMasker (Repbase) and RepeatProteinMasker^1^. Tandem repeats were identified by Tandem Repeat Finder (TRF) (Benson, 1999). The number of simple sequence repeats (SSRs) were calculated using the results of TRF according to Youssef et al. (2013). The rRNAs and tRNAs were identified using RNAmmer 1.2 (Lagesen et al., 2007) and tRNAscan-SE 1.23 (Lowe and Eddy, 1997), respectively. BLAST was used for the annotation of gene models against KEGG, GO, CAZy, Uniprot_Swissprot and non-redundant (NR) databases (Bard and Winter, 2000; Kanehisa et al., 2004; Cantarel et al., 2009; The UniProt Consortium, 2015). The genome assembly and gene calling and annotation were conducted by BGI (Shenzhen, China). The raw data was submitted to SRA under the accession number: PRJNA517297.

### RNAseq Mapping and Differentially Expressed Gene Analysis

The RNA for RNAseq analysis were isolated from the mid- and late-growth stages of the anaerobic fungal monoculture and co-culture. The RNAseq libraries, which included only mRNA, were generated according to the Illumina TruSeq RNA sample protocol. The mRNA was enriched using oligo-dT (Rio et al., 2010). Paired-end sequencing was conducted on an Illumina Hiseq 2000 platform (BGI, Shenzhen, China). All quality-filtered reads were mapped to the genome and genes by BWA (Li and Durbin, 2009) and Bowtie (Langmead et al., 2009), respectively. The number of reads produced per sample and the mapping results are provided in Supplementary Table 2. The quantification of gene expression was calculated in fragments per kilobase of transcript per million mapped reads (FPKM) with the RSEM package (Li and Dewey, 2011). To assess variability between biological replicates, the coefficient of determination *R*^2^ was calculated between biological replicate pairs using RSEM-generated FPKM values (Supplementary Table 3). The raw data was submitted to SRA under the accession number: PRJNA517315. Differentially expressed genes were screened with the NOISeq package (Tarazona et al., 2015) according to the following criteria: fold change > ±2 and divergence probability >0.8.

### Isobaric Tags for Relative and Absolute Quantization (iTRAQ) Analysis of Proteins

Proteins for iTRAQ analysis were collected from the mid- and late-growth stages of the anaerobic fungal monoculture and co-culture. The cells were digested and labeled according to Yan et al. (2016). One biological replicate from each sample (four samples in total) was then mixed as one iTRAQ set resulting in three iTRAQ sets that were analyzed. The mixed fractions were then separated by liquid chromatography (LC) and analyzed by two-step mass spectrometry (MS) (Yan et al., 2016). All procedures were conducted at BGI (Shenzhen, China). The MGF files, converted from the raw data using a 5600 msconverter, were used for protein identification with the Mascot search engine (Matrix Science, London, United Kingdom; version 2.3.02) against the fungal transcriptome containing 17,639 sequences (Yan et al., 2016). The identification of proteins in the three sets is shown in Supplementary Table 4. The proteomic dataset was deposited in the iPROX database under the accession number IPX0001499000. The criteria for differential expression of proteins was a *P-*value < 0.05 and fold change > ±1.2 in at least two iTRAQ sets.

### Nucleotide Sequencing of 28S rRNA Gene and ITS Sequences and Phylogenetic Analysis

The genomic DNA from the anaerobic fungal monoculture was used to amplify the 28S rRNA gene using the primer pair AF-LSU (5′-GCTCAAAYTTGAAATCTTMAAG-3′) and AF-LSU (5′-CTTGTTAAMYRAAAAGTGCATT-3′) (Dollhofer et al., 2016). To amplify the ITS sequence, primers ITS1 (5′-TCCGTAGGTGAACCTGCGG-3′) and ITS4 (5′-TCCTCCGCTTATTGATATGC-3′) were used (White et al., 1990). The PCR reaction (20 μl) consisted of 0.5 μl of each primer, 1 μl of the template DNA and 10 μl of PCR Master Mix. For 28S rRNA gene amplification, after initial denaturation at 94°C for 3 min, 36 cycles of amplification were performed, with 94°C for 20 s denaturation, 61°C for 45 s annealing, 72°C for 45 s extension, and a final extension of 72°C for 10 min. For the amplification of ITS sequence, we performed an initial denaturation at 95°C for 3 min, followed by 39 cycles of amplification with 95°C for 30 s denaturation, 52°C for 1 min annealing, 72°C for 1 min extension, and a final extension of 72°C for 5 min. The sequences were deposited at the GenBank under accession numbers MG250475 and MG250482 for 28S rRNA gene and ITS sequences, respectively. 28S rRNA gene and ITS sequences from representatives of the anaerobic fungal genera were retrieved and used to construct phylogenetic trees with MEGA 6 (Tamura et al., 2013).

### Chemical Analysis

The head-space gas of the culture was collected and analyzed for relative abundances of H_2_ and CH_4_ using GC-TCD (Agilent 7890B, Agilent, Santa Clara, CA, United States) according to Li et al. (2016). The volumes of H_2_ and CH_4_ were then calculated according to the total gas production. The concentration of ethanol was measured by GC-TCD using a method described by Li et al. (2016). The concentrations of formate, acetate, lactate, malate, citrate, and succinate were analyzed by HPLC (1220 Infinity LC system, Agilent, Santa Clara, CA, United States) with a reversed phase column ZorbaxSB-Aq (Agilent, Santa Clara, CA, United States) according to Li et al. (2016). The statistical analysis of glucose, gas, pH, and fermentation end products was conducted in RStudio^2^ and a significant effect was declared at *P* < 0.05.

## Results

### The Genome of Anaerobic Fungus *Pecoramyces* sp. F1

The anaerobic fungus in the present study was isolated in co-culture with the methanogen, *M. thaueri* and the results were published by Jin et al. (2011). Based on the fungal morphology, particularly the monocentric fungal thallus and presence of monoflagellated zoospores, the fungal component of the co-culture was assigned to the genus *Piromyces* (Jin et al., 2011). Subsequently it became apparent that the newly discovered genus, *Pecoramyces* was morphological similar to some *Piromyces* isolates (Hanafy et al., 2017). To obtain a more accurate identification of our fungal isolate, we applied molecular techniques based on the amplification and sequencing of the gene encoding the 28S rRNA and its ITS sequences. Using these sequences information, two phylogenetic trees were constructed based on the 28S rRNA gene sequence and the ITS sequence, respectively (Supplementary Figure 2). Both phylogenetic trees confirmed that the fungus isolated in co-culture with *M. thaueri* (Jin et al., 2011) is a member of the newly described anaerobic fungal genus, *Pecoramyces* (Hanafy et al., 2017), and is subsequently referred to as *Pecoramyces* sp. F1.

The genome of *Pecoramyces* sp. F1 was sequenced using paired-end Illumina technology with approximately 268× coverage. Results estimated the genome size of this fungus to be 106.83 Mb (Table 1). As observed in previously reported anaerobic fungal genomes (Youssef et al., 2013; Haitjema et al., 2017), *Pecoramyces* sp. F1 exhibited low GC content (16.07%) with a very low proportion of the genome used in coding for proteins (23.54%). From the data, it was estimated that the genome encoded 17,740 genes with an average length of 1,918 bp. A comparison of the *Pecoramyces* sp. F1 genome with five published anaerobic fungal genomes is shown in Table 2. The implications relating to genome structure are discussed later. The putative pathway for metabolism of glucose by *Pecoramyces* sp. F1 was demonstrated in Supplementary Figure 3 based on genomic and transcriptomic data. Comparison of the genomes of anaerobic fungi and aerobic fungi was demonstrated in Supplementary Figure 4.

**Table 1:** The assembly of the genome of *Pecoramyces* sp. F1.

Items	Scaffold	Contig
Total number	10,442	19,426
Total length (bp)	106,834,627	98,707,616
N50 (bp)	40,524	10,106
N90 (bp)	2,916	2,011
Max length (bp)	272,868	156,300
Min length (bp)	1,000	200
Sequence GC (%)	16.07	16.07


*Three libraries with insert sizes of 170 bp, 350 bp, and 6,000 bp were prepared. A total of 28.67 Gb in 159,302,966 quality-filtered paired-end reads was used for assembly with SOAPdenovo V1.05 using a kmer value of 43.*

**Table 2:** A comparison of genomes of *Pecoramyces* sp. F1 and other anaerobic fungi.

Items	*Pecoramyces* sp. F1	*Pecoramyces ruminantium*^#^	*Piromyces* sp. E2^$^	*Piromyces finnis*^$^	*Anaeromyces robustus*^$^	*Neocallimastix californiae*^$^
Estimated genome size (Mb)	106.83	100.95	71.02	56.46	71.69	193.03
Number of scaffolds	10,442	32,574	1,656	232	1035	1,819
Protein coding (%)	23.54	20.60	23.90	30.35	27.41	15.22
Number of genes	17,740	16,347	14,612	11,314	13,081	21,028
Average gene length (bp)	1,918	1,623	1,675	2,278	2,350	2,216
Number of exons	66,993	52,044	45,130	54,796	60,136	86,802
GC content (%)	16.07	17.00	21.80	21.18	16.30	18.20


*^#^The data were reported by Youssef et al. (2013).*^$^*The data were reported by Haitjema et al. (2017).*

### Effect of Co-culturing With a Methanogen on the Metabolism of *Pecoramyces* sp. F1 at Mid-Growth Stages

**FIGURE 1:**
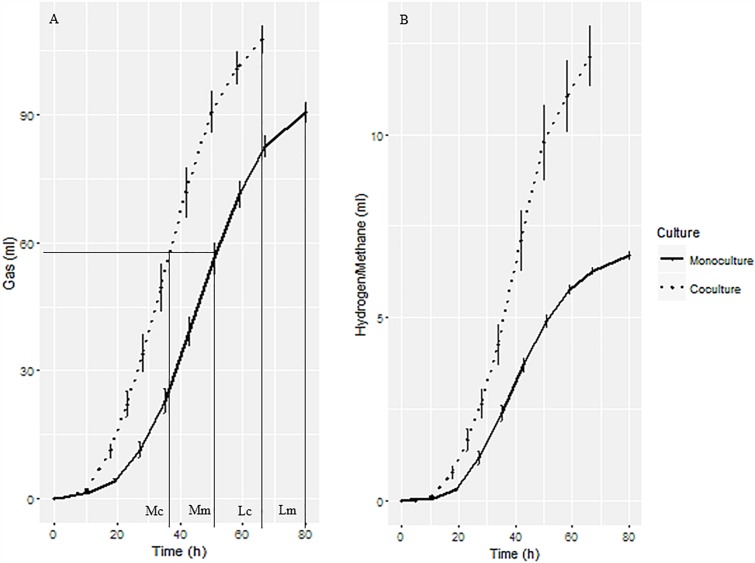
Gas production of anaerobic fungal monoculture and fungal-methanogen co-culture. **(A)** Cumulative gas production curve showing the sampling time for transcriptomic, proteomic, and fermentation end products analysis. Mc, mid-growth stage of co-culture; Mm, mid-growth stage of monoculture; Lc, late-growth stage of co-culture; Lm, late-growth stage of monoculture. **(B)** H_2_ and CH_4_ production from fungal monoculture and co-culture, respectively.

The gas production curves of the anaerobic fungal monoculture and co-culture with the methanogen are shown in Figure 1. The co-culture grew more rapidly and produced more gas, reaching mid- and late-growth stages sooner than the corresponding axenic cultures (Figure 1A). A total gas volume of 107 ml in the anaerobic fungus/methanogen co-culture was measured after 66 h of cultivation compared with 90 ml after a longer incubation time of 80 h of the monoculture. Large amounts of H_2_ accumulated in the monoculture whereas it was undetectable in the co-culture. As expected, CH_4_ accumulated in the anaerobic fungus/methanogen co-culture (Figure 1B). For further molecular analysis, samples were taken at mid- and late-growth stages.

Based on transcriptional analysis (mRNA data), at the mid-growth stage 12,262 ± 171 and 12,176 ± 311 genes were expressed in the monoculture and co-culture, respectively (*P* > 0.05). In comparison to the monoculture, it was observed that 62 and 121 genes were up-regulated and down-regulated, respectively, in the co-culture (Supplementary Table 5). The top 10 up-regulated and down-regulated genes and their functional annotations are shown in Table 3. Half of the top 10 up-regulated genes were annotated as fiber-degrading enzymes. The number of genes undergoing alternative splicing were examined and 8,281 ± 878 and 7,727 ± 169 alternatively spliced genes were detected in the monoculture and co-culture, respectively (*P* > 0.05).

**Table 3:** The top 10 up-/down-regulated genes of anaerobic fungus *Pecoramyces* sp. F1 at mid- and late-growth stages.

Stages	Up-regulated genes	Annotation (NCBInr)	Dow-regulated genes	Annotation (NCBInr)
Mid-growth stage	A07452	–	A15543	–
	A03863	–	A06045	–
	A11553	–	A03640	PREDICTED: LRR receptor-like serine
	A14137	Hypothetical protein	A14257	F5/8 type C domain protein, partial
	A04599	Sugar transporter	A18279	Extracellular alpha amylase
	A00805	Aldo/keto reductase diketogulonate reductase	A03239	Chitin binding protein, partial
	A14029	Putative cellulase	A16618	Rubrerythrin
	A06074	Cellobiohydrolase II-like cellulase CelI	A17342	Circumsporozoite protein
	A08689	Putative cellulase	A16337	Conserved hypothetical protein
	A06592	Putative cellulase	A06176	–
Late-growth stage	A15892	Hypothetical protein Haur_1598	A14983	–
	A08101	Lectin-B	A18355	–
	A15439	PREDICTED: CCR4-NOT transcription complex subunit 1-like	A11982	Putative uncharacterized protein
	A00782	Hypothetical protein Haur_1598	A12645	Endo-1,3-1,4-beta-glucanase
	A01657	Hypothetical protein PFL1_01810	A10479	Cellulase
	A12240	Conserved hypothetical protein	A13908	Hypothetical protein BATDEDRAFT_27702
	A03105	Circumsporozoite protein	A13113	Beta-glucosidase
	A00753	Hypothetical protein RO3G_04189	A05614	Alpha-amylase
	A15083	Hypothetical protein Haur_1598	A14764	Pyruvate kinase, partial
	A10600	Circumsporozoite protein	A06138	Endoglucanase B


In addition to the transcriptional analysis, proteomic analysis was carried out on total proteins at mid- and late-growth stages using the iTRAQ approach. A total of 2,149 proteins were identified (MASCOT) and quantified in all three replicates in both cultures at the mid- and late-growth stages. In comparison with the monoculture, it was observed that 117 and 162 proteins were up-regulated and down-regulated, respectively, in the co-culture at the mid-growth stage. The top 10 up-regulated and down-regulated proteins and their functions are shown in Table 4. It is significant that many of the transcripts and proteins that were highly up-regulated or down-regulated had no matches in the databases included in this study (Tables 3, 4), however, a large number of proteins associated with cellular-binding and transmembrane activities were moderately up-regulated (>2 and <100 folds) (Supplementary Table 6).

**Table 4:** The top 10 up-/down-regulated proteins of anaerobic fungus *Pecoramyces* sp. F1 at mid- and late-growth stages.

Stages	Up-regulated proteins	Annotation (Uniprot_Swissprot)	Dow-regulated proteins	Annotation (Uniprot_Swissprot)
Mid-growth stage	A10870	–	A08105	Endochitinase A
	A11918	Ubiquinone/menaquinone biosynthesis methyltransferase ubiE	A16996	Probable isoprenylcysteine alpha-carbonyl methylesterase ICMEL1
	A10968	–	A00985	Glutaredoxin-C1
	A01230	–	A04792	Mannan endo-1,4-beta-mannosidase B
	A17203	–	A11779	26 kDa endochitinase 1
	A15978	Uncharacterized symporter ynaJ	A15646	Enamine/imine deaminase
	A11958	ATP-binding cassette sub-family A member 1	A11651	–
	A08103	D-Xylose-proton symporter	A07589	Transcriptional activator HAP5
	A14498	ABC transporter A family member 1	A12393	–
	A15678	–	A04227	Zinc-type alcohol dehydrogenase-like protein PB24D3.08c
Late-growth stage	A15978	Uncharacterized symporter ynaJ	A04324	60S ribosomal protein L27a (fragment)
	A01843	Uncharacterized symporter ynaJ	A12393	–
	A08103	D-Xylose-proton symporter	A09942	Peptidyl-prolyl *cis*–*trans* isomerase pin1
	A10870	–	A08105	Endochitinase A
	A06767	–	A18633	–
	A07467	Extracellular matrix protein FRAS1	A04839	Adenine phosphoribosyltransferase
	A01230	–	A07269	Guanylate kinase
	A14498	ABC transporter A family member 1	A06738	Histidinol-phosphate aminotransferase
	A06268	Tubulin-specific chaperone A	A12012	–
	A17203	–	A15624	–


**FIGURE 2:**
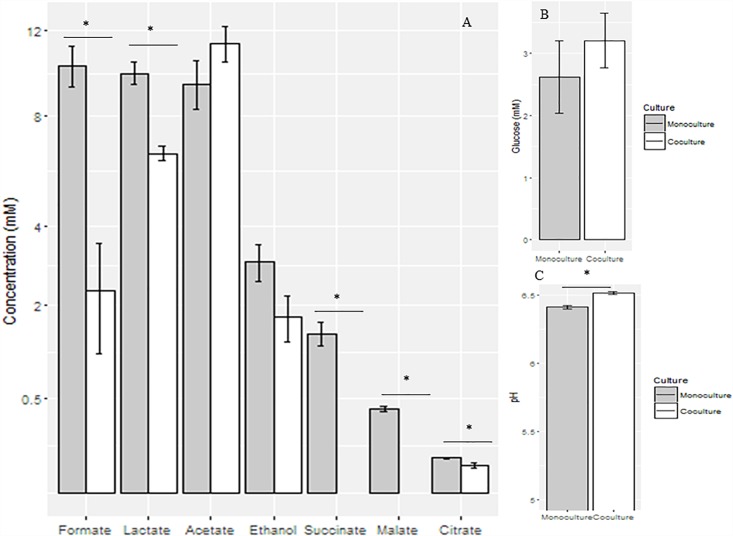
Concentrations of fermentation end products **(A)** and glucose **(B)** and pH values **(C)** in the supernatant of anaerobic fungal monoculture and co-culture at the mid-growth stage. ^∗^Means significant difference between two groups.

The pH value of the co-culture, although not very different, was significantly higher (6.5 ± 0.03) than that of the monoculture (6.4 ± 0.03) (*P* < 0.05) at the mid-growth stage. Metabolites, including formate, lactate, acetate, ethanol, succinate, malate, and citrate were detected in the supernatant of the monoculture; formate, lactate, succinate, malate, and citrate concentrations were significantly decreased when measured in the anaerobic fungus/methanogen co-culture when compared with the fungal monoculture (Figure 2).

The effects of the methanogen on the metabolism of *Pecoramyces* sp. F1 in co-culture at the mid-growth stage is presented in Supplementary Figure 5. The expression levels of aconitase and NADH dehydrogenase genes were down-regulated in the co-culture, while no significant differences were observed at the protein level. The expression levels of lactate dehydrogenase and pyruvate formate lyase (PFL) genes were not affected by co-culturing the fungus with the methanogen, although they were up-regulated at the protein level. In the case of aldehyde/alcohol dehydrogenase, it was found to be down-regulated at both the transcription and protein levels.

### Effect of Co-culturing With a Methanogen on the Metabolism of *Pecoramyces* sp. F1 at Late-Growth Stages

Measurements made at the late-growth stage showed that 11,978 ± 237 and 10,010 ± 348 genes were expressed in the monoculture and co-culture, respectively (*P* < 0.05). Relative to the monoculture, 42 and 852 of the expressed genes were up-regulated and down-regulated, respectively, in the co-culture. It was observed that most of the highly up-regulated genes at the transcriptional level in the co-culture (RNA fold change > ±100) were related to binding activities in the cell (Supplementary Table 7). The top 10 up-regulated and down-regulated genes and their functional annotations are shown in Table 3. In comparison to the mid-growth stage, at the late-growth stage, fewer genes were alternatively spliced. Thus, we observed 5,908 ± 603 and 2,061 ± 226 genes were alternatively spliced in the monoculture and the co-culture, respectively (*P* < 0.05).

In the late-growth stage, the number of proteins up-regulated was double that at the mid-growth stage cultures (276 versus 117). In the case of the down-regulated proteins, however, there was no difference in the numbers observed for the mid- and late-growth stage cultures (168 versus 162). Most of the highly up-regulated proteins (protein ratio > ±2) were related to sporulation, transmembrane, and cellular-binding activities (Supplementary Table 8). The top 10 up-regulated and down-regulated proteins and their functions are shown in Table 4.

**FIGURE 3:**
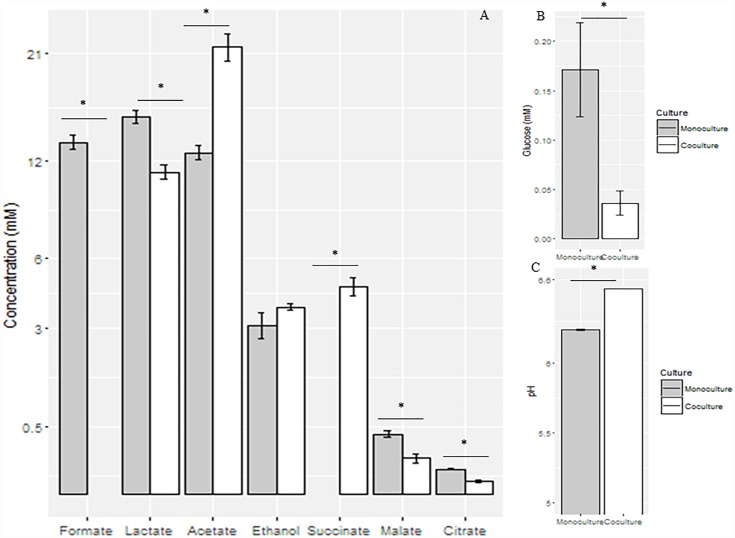
Concentrations of fermentation end products **(A)** and glucose **(B)** and pH values **(C)** in the supernatant of anaerobic fungal monoculture and co-culture at the late-growth stage. ^∗^Means significant difference between two groups.

As observed in the mid-growth stage, the pH value of the co-culture (6.53 ± 0.002) was significantly higher than that of the monoculture (6.24 ± 0.01) (*P* < 0.05) at the late-growth stage. Relative to the monoculture, the concentrations of formate, lactate, malate, and citrate were significantly decreased in the co-culture (*P* < 0.05), while the concentrations of acetate and succinate were significantly increased in the co-culture (*P* < 0.05). In contrast, the concentration of ethanol did not vary between the monoculture and the co-culture (Figure 3).

The effects of co-culturing the methanogen with *Pecoramyces* sp. F1 on metabolism at the late-growth stage are shown in Supplementary Figure 6. At the gene expression level, all of the enzymes, except for fumarase, involved in the metabolism of glucose in the anaerobic fungus were down-regulated when cultured with the methanogen, while only glyceraldehyde-3-phosphate dehydrogenase (GAPDH), phosphoglycerate mutase (PGM), PFL, and aldehyde/alcohol dehydrogenase (ADH) were also down-regulated at the protein level.

### Effect of Co-culturing With a Methanogen on the Expression of Carbohydrate-Targeting Enzymes of *Pecoramyces* sp. F1 at the Mid- and Late-Growth Stages

The top 20 differentially expressed fiber-degrading enzymes at the mid- and late-growth stages were examined (Table 5). Importantly, all of these genes were down-regulated at the late-growth stage. Although it was not anticipated that polysaccharide-degrading enzymes are required for the metabolism of glucose, our search for the top 20 fiber-degrading enzymes showed that several genes coding for such enzymes were up-regulated in the mid-growth stage. The up-regulated genes included the afore-mentioned putative cellulase and others with encoded polypeptides annotated as cellulases, endoxylanases, alpha-amylases and a feruloyl esterase. Thus, a broad range of polysaccharide-degrading enzymes were released during the early stages of glucose metabolism in co-culturing of *Pecoramyces* sp. F1 with its syntrophic methanogen. However, these polysaccharide targeting enzymes were down-regulated during the late-growth stage.

**Table 5:** Top 20 differentially expressed fiber degrading enzymes at mid- and late-growth stages.

Gene ID	Mid-growth stage^#^	Late-growth stage	Annotation (NCBInr)
A16134	5.57	-7.07	Putative cellulase
A11665	1.86	-8.19	Alpha-amylase
A13113	-0.03	-9.81	Beta-glucosidase
A16053	5.82	-3.81	Putative cellulase
A18358	0.17	-9.45	AmyE alpha-amylase
A07248	-0.66	-9.61	Feruloyl esterase
A17896	-0.07	-8.51	Endo-1,4-beta-xylanase
A17801	2.11	-6.20	Alpha-amylase G-6
A02885	4.94	-3.27	Endoxylanase
A14736	2.63	-5.03	Pectate lyase D
A07671	-0.52	-8.13	Cellulase Cel48A precursor
A03809	1.09	-6.49	Alpha-amylase MalS
A11825	4.54	-2.81	Putative cellulase
A10479	-2.64	-9.98	Cellulase
A16521	3.54	-3.66	Putative cellulase
A02179	4.09	-2.81	Putative cellulase
A17482	4.22	-2.59	Glucan endo-1,3-beta-D-glucosidase
A09998	0.44	-6.36	1,4-beta-D-glucan-4-glucanohydrolase
A08700	0.66	-6.09	Alpha-amylase
A07525	5.17	-1.55	Putative cellulase


*^#^The values are log_*2*_ of (co-culture FPKM/monoculture FPKM).*

## Discussion

Isolation and maintenance of anaerobic fungi requires a relatively complex, strictly anaerobic culture methodology limiting their study to relatively few research groups world-wide. Consequently, we do not have a good understanding of the diversity and taxonomy of these unique fungi. According to classical taxonomy, zoospore ultrastructure and to a lesser extent, fungal morphology were used to assign generic and specific names to isolates (Theodorou et al., 1996; Ozkose et al., 2001). More recently, molecular techniques based on the amplification and sequencing of genes encoding the 28S rRNA gene and ITS sequences have been used to aid classification. In the current work, molecular techniques were used to reassign the isolate *Piromyces* sp. F1 to *Pecoramyces* sp. F1. To date, only one species of *Pecoramyces* (*P. ruminantium*) has been described (Youssef et al., 2013; Hanafy et al., 2017). To date, a limited number of publications have studied fungal/methanogen interactions (Mountfort et al., 1982; Nakashimada et al., 2000; Jin et al., 2011; Li et al., 2016). In much of the original work, fungi and methanogens were isolated separately from different ruminal environments (Mountfort et al., 1982; Nakashimada et al., 2000). In working with the new isolate of *Pecoramyces* and its syntrophically associated methanogen, *M. thaueri*, we studied the metabolism of this isolate on glucose to obtain primary information about a *Pecoramyces* strain grown in monoculture and in co-culture with a syntrophic methanogen.

The estimated genome size of *Pecoramyces* sp. F1 matched that of the previously reported estimate for *Pecoramyces ruminantium*, as shown in Table 2 (Youssef et al., 2013). This observation shows that *Pecoramyces* has a larger genome size compared with the *Piromyces* and *Anaeromyces* genera, although the estimated genome size of a *Neocallimastix* is double the size of *Pecoramyces*. In contrast, the data further demonstrates that the anaerobic fungal genomes are consistently AT-rich (GC% content range from 16 to 22; Table 2). The genera *Piromyces* and the *Anaeromyces* appear to have fewer genes (∼13,000) compared to the genus *Pecoramyces* (∼17,000), while the gene number reported for *Neocallimastix* is almost twice that for *Piromyces* and *Anaeromyces*. While *Piromyces finnis* codes for approximately 11,000 genes, the number of genes coded by *Piromyces* sp. E2 is not very different from that of *Pecoramyces ruminantium* (Youssef et al., 2013). The number of genes coded by *Neocallimastix californiae* (Haitjema et al., 2017) shows that this fungal species uses about twice the genome of the *Pecoramyces* strains to encode a number of genes only slightly higher than that of the *Pecoramyces* strains. Therefore, the protein coding percentage of the genome of the reported *Neocallimastix* strain is very low in comparison with the other genera discussed in this manuscript.

The results from this study show that alternative splicing occurs in *Pecoramyces* sp. F1, as reported in the aerobic fungi (Grutzmann et al., 2014). Meanwhile, the average alternative splicing rates of *Pecoramyces* sp. F1 (∼45% and 22% at the mid- and late-growth stages, respectively) seem higher than the aerobic fungi, which was 6.4% on average (Grutzmann et al., 2014). Furthermore, it was observed that alternative splicing in the co-culture of the fungus with the methanogen was significantly lower than the fungal monoculture at the late-growth stage. The decreased splicing might be due to the limitation of the substrate in the culture or a slower growth rate associated with substrate depletion; Birch et al. (1995) reported that differential splicing in *Phanerochaete chrysosporium* might regulate the specificities of substrate of this fungus.

As observed in previous reports (Cheng et al., 2009; Jin et al., 2011; Li et al., 2016) during co-culturing of anaerobic fungi with methanogens, total gas production exceeded that of the gut fungal culture alone and the rate of gas production was faster. This observation confirms the increased efficiency with which the anaerobic fungi ferment substrates in the presence of the hydrogen-utilizing methanogen. Sampling at the mid-growth stage showed that total mRNA expression was not different between the monoculture and co-culture and the number of up-regulated genes was half the number of the down-regulated genes. However, by the late-growth phase, mRNA expression of the co-culture was significantly lower than that of the monoculture and the ratio of the up-regulated and down-regulated genes were dramatically decreased. The mRNA expression profiles suggest that on encountering a glucose energy source, *Pecoramyces* sp. F1 secretes a large number of polysaccharide degrading enzymes including endoglucanases, chitinases, amylases, and licheninases. In the case of the monoculture, this enzyme secretion appears to continue throughout growth, perhaps due to comparatively inefficient substrate utilization. On coupling the fermentation of the anaerobic fungus with the methanogen, the efficiency of the fermentation increased, leading to a down-regulation of the expression of the polysaccharide degrading enzymes. As shown in Table 5, this is particularly so for the putative enzymes involved in cellulose metabolism, including about six putative cellulases, likely reflecting the hydrolysis of the cellulose backbone. The efficiency of the fermentation in anaerobic fungus/methanogen co-culture increases is likely due to the removal of H_2_ through interspecies transfer to the syntrophic methanogen to produce CH_4_. In the fungal cell, the oxidization of NADH into NAD^+^ and H^+^ is associated with the production of acetic acid. This pathway is likely to be favorable for obtaining higher amounts of ATP, compared with the more reduced electron sinks end-products (e.g., lactate, ethanol) used by anaerobic fungi to regenerate NAD^+^ for glycolysis (Bauchop and Mountfort, 1981; Marvin-Sikkema et al., 1990).

The results in the present study are in agreement with the observation that during syntrophic interactions between several ruminal organisms with hydrogen-removing methanogens, a shift in the metabolism occurs leading to extra ATP gain by the organism co-cultured with the methanogen (Bauchop and Mountfort, 1981; Marvin-Sikkema et al., 1990). Unlike the transcriptomic data, major shifts in the proteomic data were not observed in the present study. This may be due to the fact that the proteins in the cell have a much longer lifetime than that of the mRNAs. A genome-wide study showed that the lifetime of mRNAs in *Escherichia coli* were between 3 and 8 min (Bernstein et al., 2002). However, the rate of intracellular protein degradation in *E. coli* was 4 h (Koch and Levy, 1955).

The changes in the metabolites observed in the present study are similar to the results observed in our previous studies (Li et al., 2016, 2017). In brief, the pH value increased significantly at the late-growth stage as formate was utilized by the co-cultured methanogen and the lactate decreased due to a reduced demand for electron sink products for regeneration of reducing equivalents. Finally, acetate increased significantly because metabolism in the hydrogenosome became more efficient.

Combining the data reported in the present study and previous reports on anaerobic fungi and methanogens co-culture (Cheng et al., 2009; Li et al., 2016, 2017), we found that in the early growth stage of the co-culture, the metabolism in the fungal cell improved and large amounts of end products were produced. At this growth stage, the substrate was adequate and only H_2_ was used by co-cultured methanogens to reduce the gas pressure, which could inhibit the microbial growth (Li et al., 2016). At the late-growth stage, the substrate was inadequate for anaerobic fungi to produce enough H_2_ and methanogens would use formate to produce methane, which increased the pH value of the culture. The metabolic interaction between the two organisms would help both of them to be competitive in the rumen. For the anaerobic fungus, the fiber-degrading ability was improved and feedback inhibition (both gas pressure and water-soluble metabolites) was eliminated. For the methanogen, it could obtain H^+^ as soon as it was produced.

In summary, in the present report we have used modern molecular approaches to assign phylogenetic placement to a new anaerobic fungal isolate and concomitantly provided a mechanistic understanding of its intermediary metabolism in co-culture with a syntrophic methanogen. We look forward to future experiments that explore interactions during degradation of more complex substrates.

## Author Contributions

YC and WZ conceived and designed the experiments. WJ, YfL, and YqL performed the experiments. YfL, YqL, WJ, YC, TS, RM, and IC generated and analyzed the data. YC, TS, RM, IC, and WZ wrote and revised the paper. All authors read and approved the final manuscript.

## Conflict of Interest Statement

The authors declare that the research was conducted in the absence of any commercial or financial relationships that could be construed as a potential conflict of interest. The handling Editor declared a past co-authorship with one of the authors YC.

## Footnotes

1 http://repeatmasker.org

2 http://www.rstudio.org
