# Supplementary material for: Combined Genomic, Transcriptomic, Proteomic, and Physiological Characterization of the Growth of Pecoramyces sp. F1 in Monoculture and Co-culture With a Syntrophic Methanogen
Source: Front Microbiol. 2019 Mar 6;10:435. doi: 10.3389/fmicb.2019.00435 (PMC6414434; doi:10.3389/fmicb.2019.00435)
Supplement: Supplementary file 2 [file Data_Sheet_2.docx]

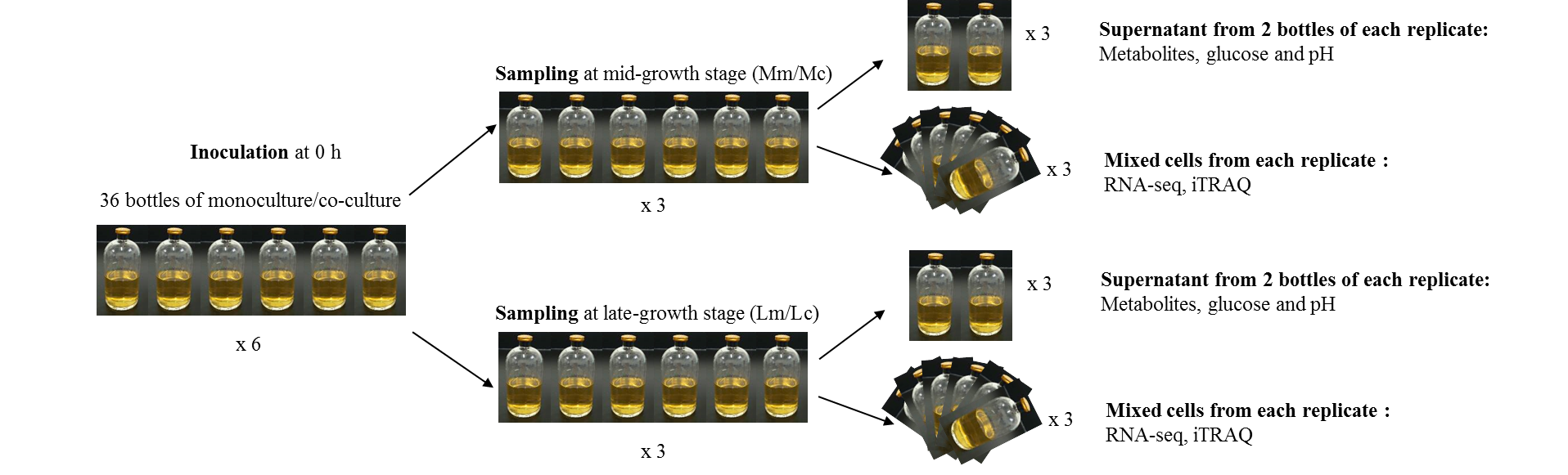


**Supplementary Figure 1.** Experimental design investigating the effects of co-cultured methanogen (*Methanobrevibacter thaueri*) on the metabolic pathways of the anaerobic fungus *Pecoromyces* sp. F1. A total of 72 bottles of anaerobic medium (36 per culture) with six replicates and six bottles for each replicate were prepared. The volume of total gas, H_2_ and CH_4_ were measured throughout the incubation using a transducer system. At the mid- and late- growth stage, samples from 3 replicates were collected for the analysis. The supernatant from 2 bottles of each replicate was collected for the analysis of glucose, pH and metabolites. The cells from 6 bottles of each replicate were mixed and split to two parts for the RNA-seq and iTRAQ analysis.


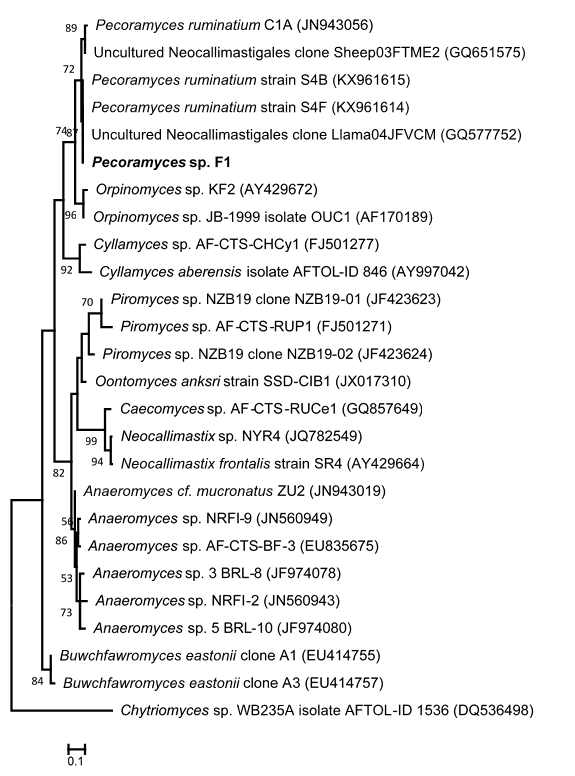

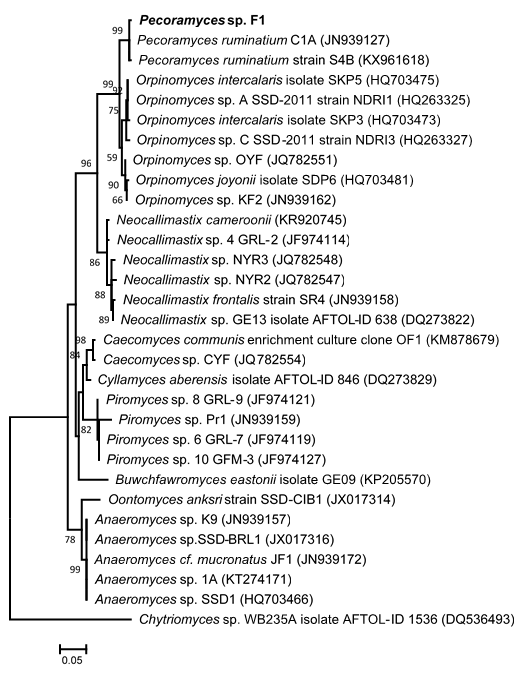


A

B

**Supplementary Figure 2.** Maximum likelihood phylogenetic trees based on 28S rRNA gene (A) and ITS (B) sequences. The aerobic fungus *Chytriomyces* sp. WB235A isolate AFTOL-ID 1536 was used to root the trees. Bootstrap values (from 500 replicates) were shown for nodes with more than 50% bootstrap support. Analysis was conducted in MEGA 6.


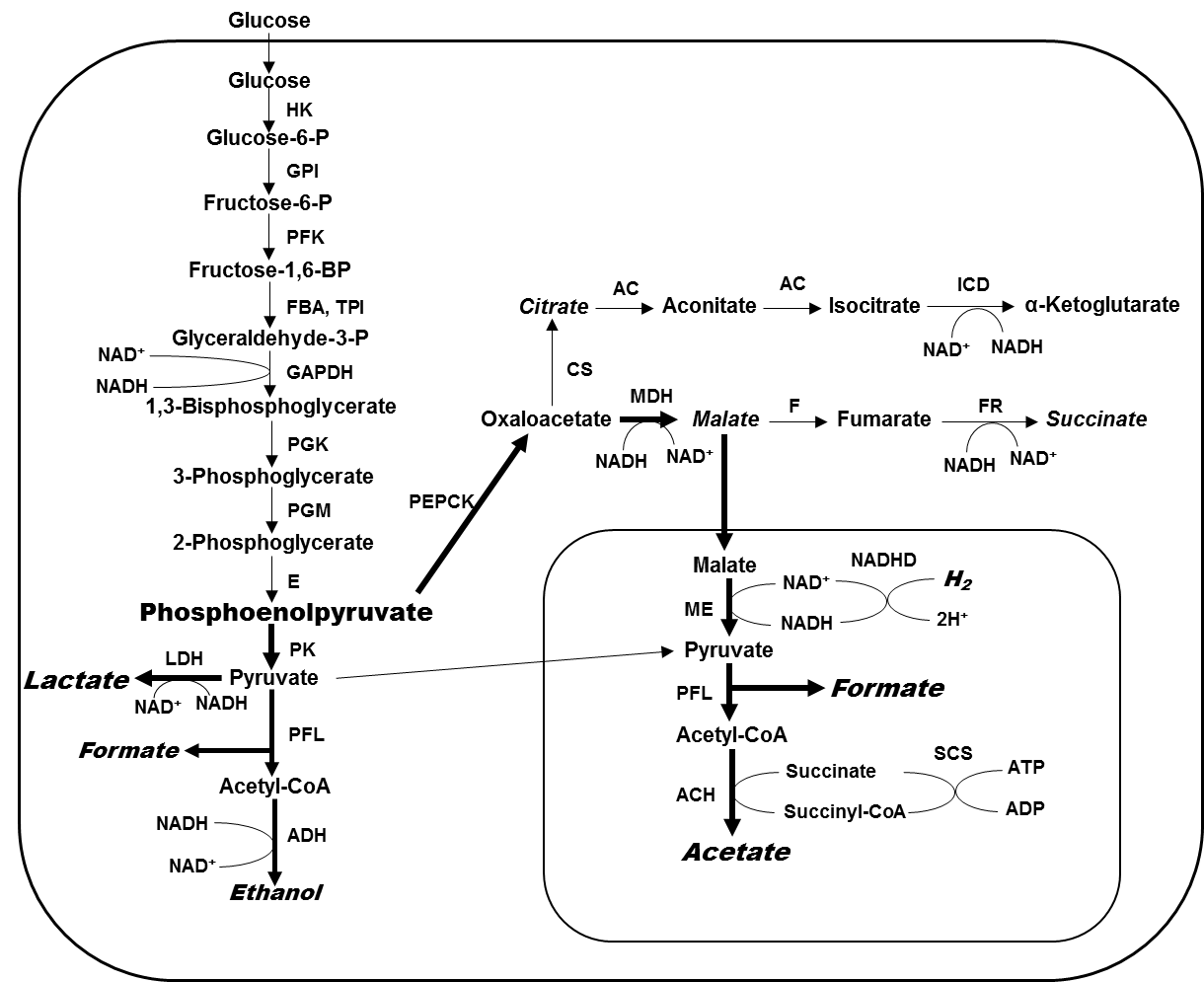


**Supplementary Figure 3.** Putative intermediate metabolic pathway of glucose by *Pecoramyces* sp. F1. The main path is indicated by bold arrows from phosphoenolpyruvate to the end metabolites. The proposed metabolites of anaerobic fungus were indicated in *Italics*. HK, Hexokinase; GPI, Glucose-6-phosphate isomerase; PFK, Phosphofructokinase; FBA, Fructose-bisphosphate aldolase; TPI, Triosephosphate isomerase; GAPDH, Glyceraldehyde-3-phosphate dehydrogenase; PGK, 3-Phosphoglycerate kinase; PGM, Phosphoglycerate mutase; E, Enolase; PK, Pyruvate kinase; PFL, Pyruvate formate lyase; ADH, Aldehyde/alcohol dehydrogenase; LDH, Lactate dehydrogenase; PEPCK, Phosphoenolpyruvate carboxykinase; MDH, Malate dehydrogenase; F, Fumarase; FR, Fumarate reductase; CS, Citrate synthase; AC, Aconitase; ICD, Isocitrate dehydrogenase; ME, Malic enzyme; NADHD, NADH dehydrogenase; ACH, Acetyl-CoA hydrolase; SCS, Succinyl-CoA synthetase.


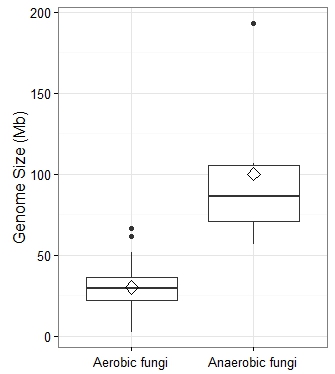

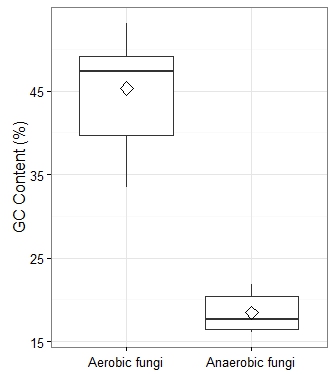

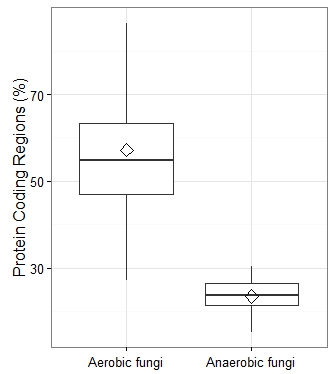


**Supplementary Figure 4.** Components of the genomes of anaerobic fungi (n=6) and aerobic fungi (n=32). The diamonds show the average values. Except for the reported *Pecoramyces* sp. F1 genome in the present study, the five anaerobic fungal genomes were reported by Youssef et al. (2013) and Haitjema et al. (2017). The 32 aerobic fungal genomes are *Neurospora crassa* OR74A (GCA_000182925.2), *Aspergillus nidulans* FGSC A4 (GCF_000149205.1), *Batrachochytrium dendrobatidis* JAM81 (GCA_000203795.1), *Aspergillus niger* ATCC 1015 (GCA_000230395.2), *Trichoderma reesei* QM6a (GCA_000167675.2), *Saccharomyces cerevisiae* S288C (GCA_000146045.2), *Schizosaccharomyces pombe* (GCA_000002945.2), *Fusarium oxysporum* f. sp. *lycopersici* 4287 (GCA_000149955.2), *Batrachochytrium dendrobatidis* JEL423 (GCA_000149865.1), *Candida albicans* SC5314 (GCA_000182965.3), *Cryptococcus neoformans* var. *grubii* H99 (GCA_000149245.3), *Sacharomyces kudriavzevii* IFO 1802 (GCA_000167075.2), *Coccidioides posadasii* C735 delta SOWgp (GCA_000151335.1), *Magnaporthe oryzae* 70-15 (GCA_000002495.2), *Trichophyton rubrum* CBS 118892 (GCA_000151425.1), *Cryptococcus gattii* VGII 2001/935-1 (GCA_000835815.1), *Aspergillus fumigatus* A1163 (GCA_000150145.1), *Aspergillus oryzae* 3.042 (GCA_000269785.2), *Histoplasma capsulatum* NAm1 (GCA_000149585.1), *Encephalitozoon cuniculi* GB-M1 (GCA_000091225.1), *Rhizoctonia solani* AG-3 Rhs1AP (GCA_000524645.1), *Rhizopus microsporus* ATCC 52813 (GCA_002708625.1), *Zymoseptoria tritici* IPO323 (GCA_000219625.1), *Coccidioides immitis* RS (GCA_000149335.2), *Rhizopus delemar* RA 99-880 (GCA_000149305.1), *Lichtheimia corymbifera* JMRC:FSU:9682 (GCA_000723665.1), *Stachybotrys chartarum* IBT 7711 (GCA_000730325.1), *Kwoniella mangroviensis* CBS 8507 (GCA_000507465.3), *Blastomyces dermatitidis* ER-3 (GCA_000003525.2), *Yarrowia lipolytica* CLIB122 (GCA_000002525.1), *Mucor circinelloides f. lusitanicus* CBS 227.49 (GCA_001638945.1), *Penicillium expansum* (GCA_000769745.1).


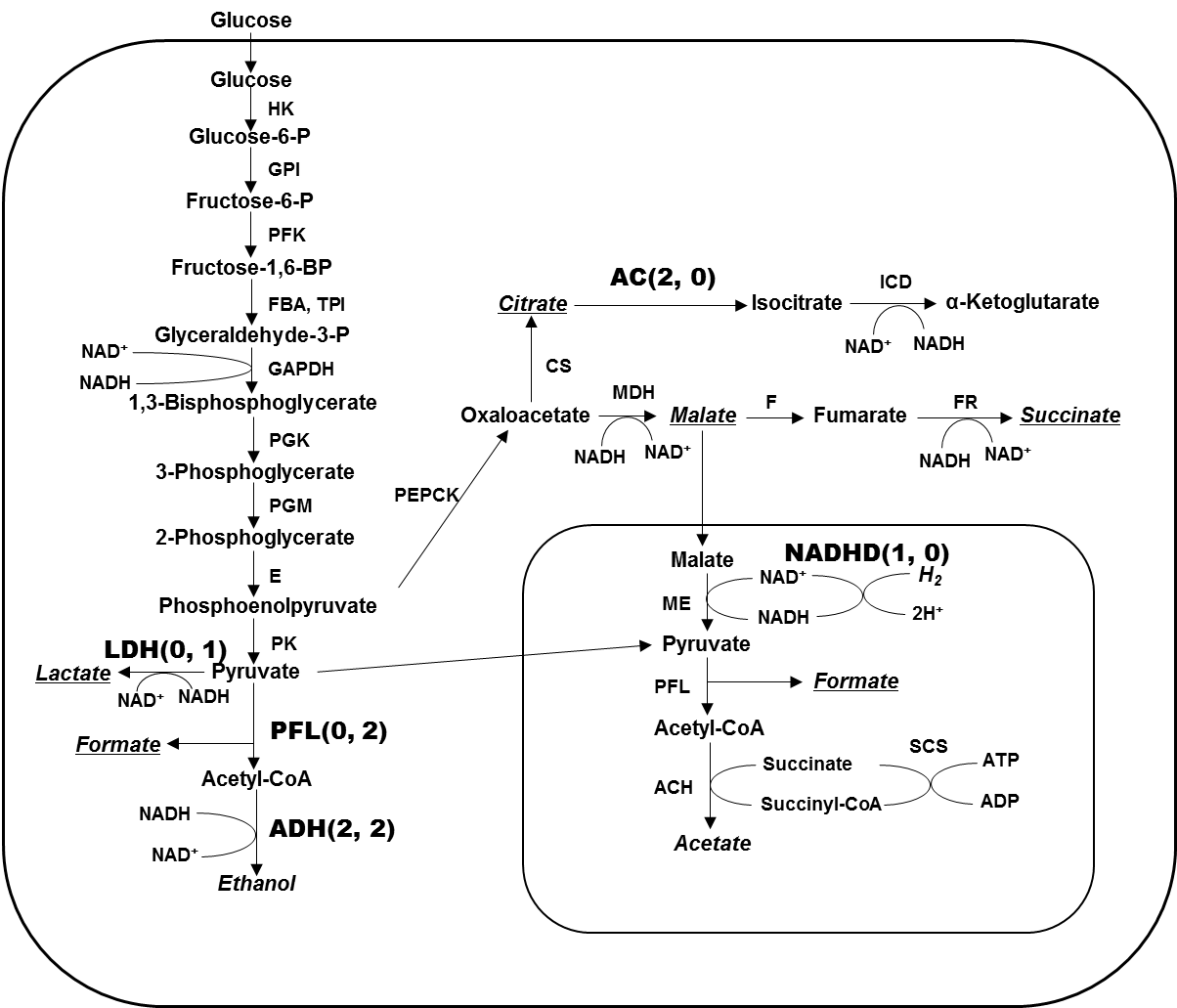


**Supplementary Figure 5.** Effects of co-culturing a syntrophic methanogen on the metabolism of an anaerobic fungus at the mid-growth stage. The number of significantly decreased genes at the RNA and protein levels are indicated in parentheses (the first number denotes RNA, the second denotes protein). The underlined metabolites were significantly decreased (*P* < 0.05).


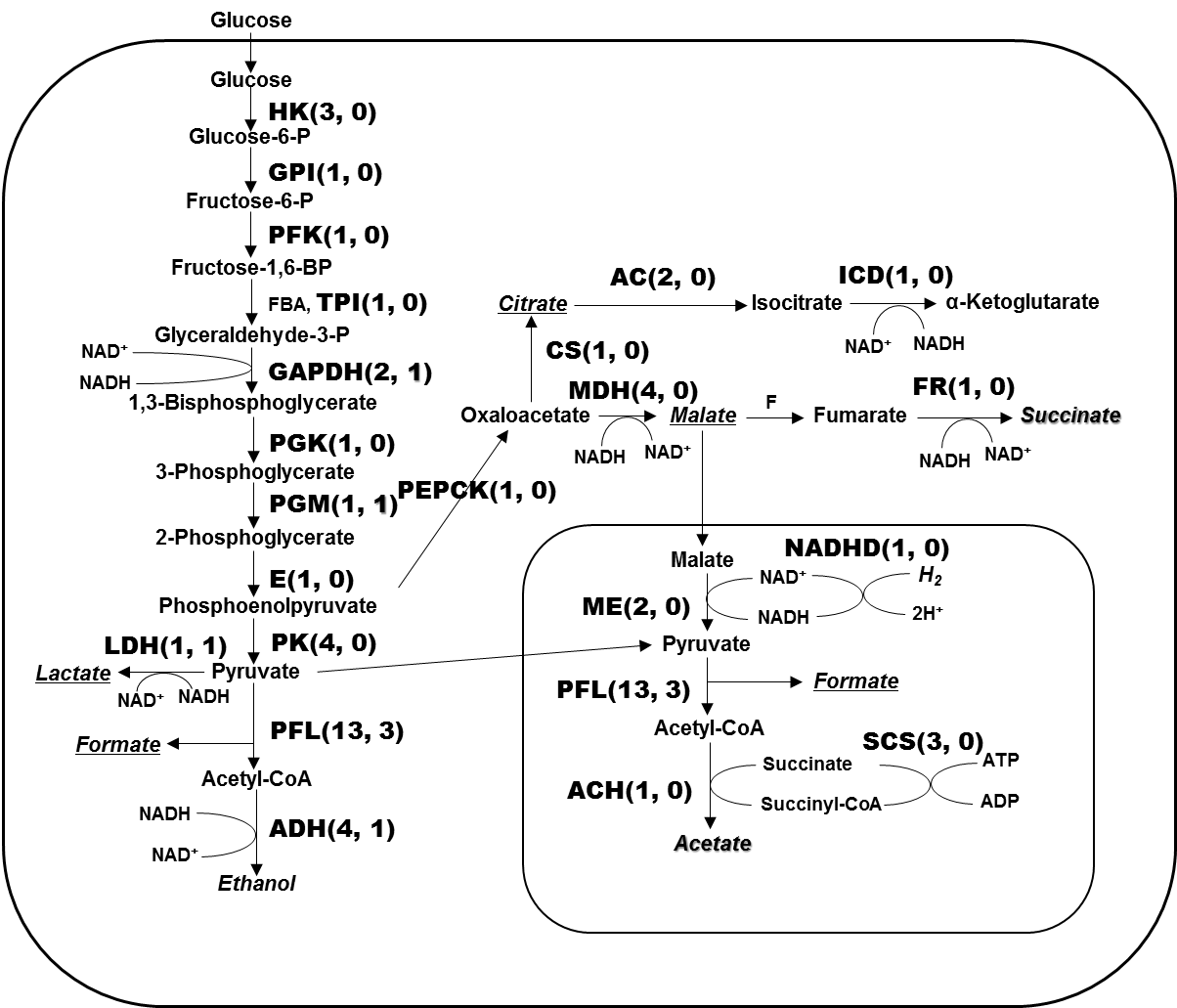


**Supplementary Figure 6.** The effect of co-culturing a syntrophic methanogen on the metabolism of an anaerobic fungus at the late-growth stage. The number of significantly changed genes at the RNA and protein levels were indicated in parentheses (the first numbers for RNA and the second numbers for protein). The underlined metabolites were significantly decreased. The metabolites marked with a shadow were significantly increased.
